# Research advances in exercise and cognitive fatigue: a bibliometric analysis and thematic strategic evolution analysis

**DOI:** 10.3389/fpsyg.2026.1809755

**Published:** 2026-07-14

**Authors:** Yeting Zhang, Jiangxi Yang, Huangyan Li, Huan Ma

**Affiliations:** 1 College of Aviation Physical Education, Civil Aviation Flight University of China, Guanghan, China; 2 College of Education Science, Sichuan Normal University, Chengdu, China

**Keywords:** bibliometrics, cognitive fatigue, executive function, knowledge mapping, sport performance

## Abstract

**Background:**

Cognitive fatigue (CF), characterized by decrements in executive function and heightened subjective exhaustion resulting from prolonged cognitive exertion, has emerged as a critical determinant of athletic performance and psychophysiological wellbeing. Despite the exponential growth in research output, systematic quantitative analyses of the intellectual structure, evolutionary trajectory, and emerging frontiers within this domain remain scarce.

**Methods:**

Drawing upon the Web of Science Core Collection and Scopus databases, this study retrieved publications addressing exercise and cognitive fatigue from 1998 to 2025 using the search strategy: TS = (“physical activity” OR exercise OR sport*) AND TS = (“mental fatigue” OR “cognitive fatigue” OR “mental fog” OR “cognitive weariness*” OR “cognitive exhaustion*”). Following systematic screening, 820 articles were included for bibliometric analysis utilizing Bibliometrix and VOSviewer.

**Results:**

Publication output rose modestly (1.84% annually), peaking in 2025 (*n* = 106), likely reflecting post-pandemic mental health research expansion and portable neurotechnology adoption. The US and China led productivity; the UK showed highest citation impact (48.16/article), suggesting influential contributions, though this may reflect publication timing and foundational works. Vrije Universiteit Brussel and University of Birmingham topped institutional output, reflecting sustained contributions within the Marcora, Meeusen, and Roelands traditions. *Frontiers in Psychology* was most influential. Keywords shifted from laboratory tasks to ecologically valid sport contexts (“football,” “team sports”). Thematic evolution moved from “chronic fatigue syndrome” to “perceived exertion” and “depression,” then to “executive function” and “combat sports”—indicating a gradual shift from descriptive symptoms to integrated cognitive-affective-physiological mechanisms. “Cognitive effort,” “physical fatigue,” and “executive function” occupied the motor themes quadrant in 2024–2025, signaling mature, structurally central topics. Findings suggest emerging brain-body-performance integration.

**Conclusion:**

This study traces an evolution from pathological fatigue measurement to executive function precision assessment. Bibliometric indicators reveal growing emphasis on cognitive effort as a motor modulator, with co-occurrence patterns identifying dual clusters around subjective perception and performance parameters. Whether this bibliographic convergence reflects validated physiological mechanisms or emerging theoretical hypotheses requires further primary experimental investigation. This analysis provides a complementary, field-level perspective that complements, rather than replaces, primary experimental research.

## 1 Introduction

Cognitive fatigue (CF), characterized by decrements in executive function, diminished motivation, and heightened subjective exhaustion resulting from prolonged cognitive exertion, has emerged as a critical determinant of human athletic performance and psychophysiological wellbeing (Smith et al., 2016). In recent years, with the advancement of scientific approaches in competitive sports and the progressive implementation of national fitness initiatives, the interactive mechanisms between exercise and cognitive fatigue have garnered increasing attention across sport science, neuroscience, and clinical medicine. Empirical evidence suggests that cognitive fatigue significantly impairs technical performance and decision-making accuracy in team sports such as football and basketball (Pan et al., 2025a), and compromises endurance performance by elevating ratings of perceived exertion (RPE) (de Lima-Junior et al., 2024). Conversely, appropriate physical activity and exercise interventions have been shown to effectively alleviate cognitive fatigue symptoms and enhance quality of life among patients undergoing cancer chemotherapy and those with multiple sclerosis (Torkhani et al., 2021; Moulton et al., 2023). Physical education (PE) settings represent a particularly salient ecological context for investigating the exercise-cognitive fatigue nexus. Unlike elite athletic environments where performance optimization is the primary objective, PE contexts involve dual-task demands wherein students must simultaneously manage motor skill acquisition, cognitive load from instructional content, and psychophysiological stress from physical exertion (Travlos, 2010). This unique configuration renders PE populations especially vulnerable to cognitive fatigue effects, as sustained attentional resources are partitioned across motor execution, tactical comprehension, and self-regulatory processes. Recent investigations have established robust associations between perceived cognitive fatigue and cognitive flexibility in sports higher education students, indicating that academic stressors compound with physical demands to produce distinct fatigue phenotypes (Karaku et al., 2024). These findings underscore that PE contexts are not merely peripheral to the exercise-cognitive fatigue domain but constitute a critical testing ground for understanding how cognitive load modulates motor efficacy in developing populations. Despite the exponential growth in research output within this domain, existing literature has predominantly focused on isolated experimental studies or narrative reviews, with a paucity of systematic quantitative analyses examining the intellectual structure, evolutionary trajectory, and emerging frontiers of the field. Bibliometrics, a scientific evaluation methodology grounded in mathematical and statistical approaches, enables the objective delineation of disciplinary developmental patterns, identification of research frontiers, and prediction of future directions through co-occurrence analysis, coupling analysis, and strategic diagram analysis (Aria and Cuccurullo, 2017). In recent years, bibliometric methodologies have been successfully applied to investigations of the traumatic brain injury–Alzheimer’s disease nexus and the gut microbiota–rheumatoid arthritis relationship, providing crucial evidence for interdisciplinary integration and theoretical innovation (Dong et al., 2023; Hu et al., 2024). Nevertheless, to date, no study has employed bibliometric approaches to conduct a systematic analysis of the exercise and cognitive fatigue domain. Consequently, the knowledge base, distribution of core research constituents, thematic evolution patterns, and emerging trends within this field remain poorly elucidated.

In light of these gaps, this study retrieved literature pertaining to exercise and cognitive fatigue spanning 1998–2025 from the Web of Science Core Collection and Scopus databases, conducting multidimensional bibliometric analyses utilizing Bibliometrix and VOSviewer. The research objectives were fourfold: (1) to delineate the spatiotemporal distribution patterns and knowledge production configurations characterizing this domain; (2) to identify influential authors, institutions, journals, and nations/regions alongside their collaborative networks; (3) to elucidate the evolutionary trajectories and strategic positioning of research themes; and (4) to examine emergent trends and prospective research directions. Through systematic knowledge mapping, this investigation seeks to furnish empirical foundations enabling researchers to comprehend domain dynamics, optimize study designs, and expand international collaborations, whilst providing theoretical grounding for the development of cognitive fatigue management protocols and performance optimization strategies.

## 2 Materials and methods

### 2.1 Data sources and search strategy

**FIGURE 1:**
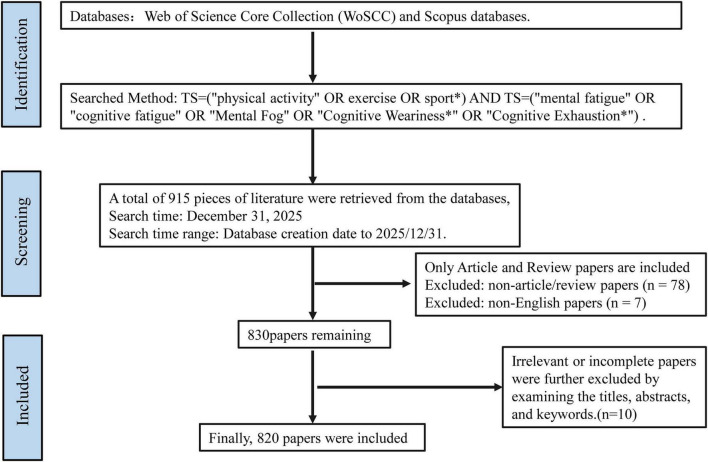
Flow diagram for the screening.

Regarding our search strategy, we selected terms such as “physical activity,” “exercise,” and “sport*” to maximize the semantic scope and align with the sports science and rehabilitation subject categories of the Web of Science/Scopus. The educational exercise context was implicitly covered by the overarching concept of “physical activity,” which encompasses structured exercise in competitive, leisure, and instructional settings. To ensure both comprehensive coverage and targeted precision in literature retrieval, this study employed a hybrid search strategy integrating MeSH terms and free-text keywords to systematically encompass core concepts and pertinent peripheral themes within the research domain. Searches were conducted simultaneously across the Web of Science Core Collection (WoSCC) and Scopus databases. The WoSCC search strategy was formulated as: TS = (“physical activity” OR exercise OR sport*) AND TS = (“mental fatigue” OR “cognitive fatigue” OR “mental fog” OR “cognitive weariness*” OR “cognitive exhaustion*”). The Scopus advanced search string was: TITLE-ABS-KEY (“physical activity” OR exercise OR sport*) AND TITLE-ABS-KEY (“mental fatigue” OR “cognitive fatigue” OR “mental fog” OR “cognitive weariness*” OR “cognitive exhaustion*”), with document types restricted to Articles or Reviews. The temporal scope spanned from database inception through December 31, 2025. Following the merging of retrieval results from both databases, a total of 915 pertinent records were identified, thereby enhancing the comprehensiveness of this systematic review (Lim et al., 2024). During data processing, two investigators independently executed literature searching, downloading, and verification procedures. Subsequently, during the screening phase, non-English publications, duplicate records, entries with incomplete bibliographic information, off-topic documents, and non-scholarly content including editorials, news items, correspondence, and conference abstracts were systematically excluded. Ultimately, 820 publications meeting the inclusion criteria were retained for analysis. The literature screening workflow is detailed in Figure 1.

### 2.2 Data analysis

This study employed the Bibliometrix R package (version 3.0.3)^1^ and VOSviewer software (version 1.6.18) for bibliometric analysis and visualization (Aria and Cuccurullo, 2017). The analytical workflow proceeded as follows: First, the R-bibliometrix package was utilized to conduct frequency analyses of publication and citation counts for authors, journals, institutions, and countries, with the top 20 entries in each category extracted. Second, synonym files (for merging equivalent terms) and deletion files (for excluding search terms and redundant keywords) were constructed to analyze temporal evolution trends in keyword frequencies from 2010 to 2025, with thematic maps generated for two distinct periods to illustrate the dynamic developmental trajectories of research themes. Additionally, the Biblioshiny web interface was employed to implement data visualization and social network mapping. VOSviewer software (version 1.6.16)^2^ was subsequently used to generate co-occurrence networks for author collaborations, countries, institutions, and keywords, wherein node size represented occurrence frequency or citation count, and connection line thickness indicated collaboration intensity. Network construction parameters were specified as follows: minimum occurrence thresholds were set at 10 for countries, authors, and institutions, 5 for journals and keywords, and 100 for document citation frequency. This integrated platform approach ensured methodological consistency and reproducibility throughout the research process (Ninkov et al., 2022; Hu et al., 2024). Unless otherwise specified, default software parameters were adopted.

## 3 Results

### 3.1 Overview of publications and time trends

**FIGURE 2:**
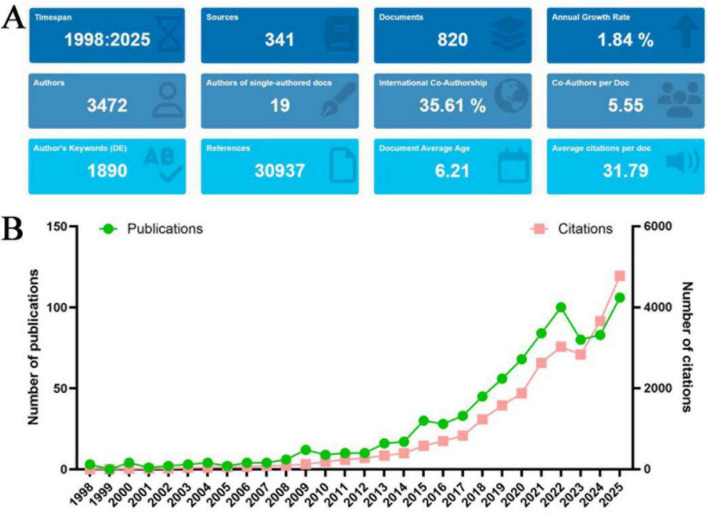
Descriptive analysis of the retrieved literature. **(A)** Main information of 820 included articles related to Exercise and CF. **(B)** Trends in the number of publications and total citations.

Following eligibility screening and quality assessment, 820 publications pertaining to exercise and cognitive fatigue were ultimately included for bibliometric analysis (see Figure 2A). These articles were authored by 3,472 researchers affiliated with 1,316 institutions across 64 countries, published in 341 journals, and cited 30,937 references from 7,303 source journals. From 1998 to 2025, annual publication output exhibited a modest upward trajectory with an annual growth rate of 1.84%. As illustrated in Figure 2B, a notable surge in publications emerged in 2015 (*n* = 30). Subsequent to 2017, the literature expanded precipitously, reaching its zenith in 2025 (*n* = 106), with this upward trend projected to persist.

### 3.2 Analysis of countries

**FIGURE 3:**
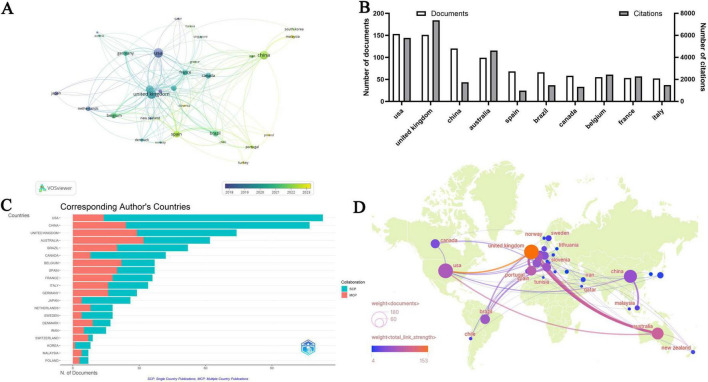
Analysis of countries. **(A)** Network visualization of countries collaborations. **(B)** The 10 leading countries ranked by publication volume. **(C)** The location of published articles based on the corresponding author’s country. **(D)** Co-authorship analysis illustrated in the geographical map.

A total of 64 countries contributed to research on exercise and cognitive fatigue. Based on corresponding author affiliations, China and the United States each produced over 220 publications, substantially outpacing other nations. Fifteen countries generated more than 10 publications, with five countries exceeding 50 research outputs. Conversely, 10 countries contributed only a single article. Analysis of corresponding author nationalities revealed that the United States dominated in publication volume with 113 articles (13.8% of total; 99 single-country publications, 14 multiple-country publications), closely followed by China with 107 articles (13.0% of total; 83 single-country publications, 24 multiple-country publications). Subsequent contributors included the United Kingdom, Australia, Brazil, Canada, Belgium, Spain, France, and Italy. Switzerland exhibited the highest proportion of multiple-country publications (77.8%), comprising 2 single-country and 7 multiple-country articles (see Figure 3C). The United Kingdom attained the highest total citation count (*n* = 7,368) and average citations per article (*n* = 48.16), whereas the United States, despite its leading publication output, demonstrated a notable citation gap relative to the United Kingdom (see Figures 3A,B). Co-authorship analysis among these nations identified the United Kingdom (TLS = 153), Australia (TLS = 98), and Spain (TLS = 82) as the three most collaborative countries (see Figure 3D).

### 3.3 Analysis of institutions

**FIGURE 4:**
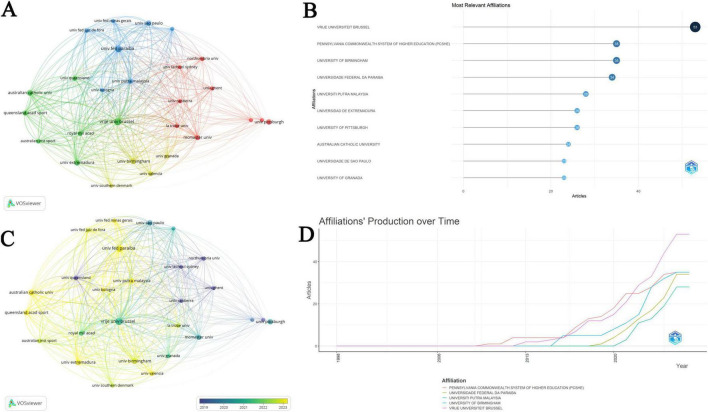
Analysis of institutions. **(A)** Network visualization of institutional collaborations. **(B)** The top 10 most contributing institutions. **(C)** The overlay visualization of bibliographic coupling between institutions. **(D)** Annual publication trends of the top five institutions.

A total of 1,316 institutions contributed publications on exercise and cognitive fatigue. Among these, 27 institutions produced more than 10 articles. Co-authorship analysis was visualized for these 27 institutions, generating network visualizations of institutional collaboration and overlay mappings of inter-institutional bibliographic coupling (Figures 4A,C). The top 10 institutions by publication output were led by Vrije Universiteit Brussel with the highest productivity (*n* = 53 articles), followed by Pennsylvania Commonwealth System of Higher Education (PCSHE) (*n* = 35) and the University of Birmingham (*n* = 35) (Figure 4B). VOSviewer-based bibliographic coupling analysis of institutions with more than 10 publications revealed that Vrije Universiteit Brussel, Universidade Federal da Paraíba, and University of Birmingham were prolific in early periods. By contrast, Universidade Federal da Paraíba, Universidad de Extremadura, and Universiti Putra Malaysia demonstrated heightened publication activity in recent years (Figure 4C). The top five institutions exhibited marked increases in article output following 2019, with Vrije Universiteit Brussel experiencing a precipitous rise and maintaining a dominant position thereafter, whilst peer institutions across Europe, North America, and Asia rapidly expanded their contributions. Post-2025, overall output stabilized, evidencing a European-led, multipolar competitive landscape (Figure 4D). Given that publication count alone provides an incomplete measure of institutional contribution, citation frequencies and total link strength (TLS) were additionally analyzed (Table 1). Collectively, Vrije Universiteit Brussel and Universidade Federal da Paraíba emerged as particularly influential contributors to this domain.

**TABLE 1:** Global output snapshot of leading institutions (publications, citations, link strength).

No.	Rank by productivity		Rank by citation impact		Rank by network centrality	
	Institution (documents)	Documents	Institution (citations)	Citations	Institution (TLS)	TLS
1	Vrije Universiteit Brussel	33	Vrije Universiteit Brussel	1,647	Vrije Universiteit Brussel	38,866
2	Universidade Federal da Paraíba	29	University of Technology Sydney	1,199	Universidade Federal da Paraíba	34,706
3	University of Birmingham	22	University of Pittsburgh	834	Universiti Putra Malaysia	21,878
4	Universidad de Extremadura	20	Ghent University	671	Royal Military Academy	20,810
5	Australian Catholic University	19	Universidade Federal da Paraíba	602	Australian Catholic University	19,185
6	University of Pittsburgh	17	University of Canberra	559	University of Birmingham	18,227
7	Universidade de São Paulo	17	The University of Queensland	551	Universidad de Extremadura	18,210
8	Universiti Putra Malaysia	16	Australian Institute of Sport	523	Universidade de São Paulo	17,282
9	McMaster University	15	Royal Military Academy	512	Queensland Academy of Sport	17,196
10	Queensland Academy of Sport	15	Universidade Federal de Pernambuco	491	Universidade Federal de Juiz de Fora	16,182

TLS, total link strength.

### 3.4 Analysis of journals

**FIGURE 5:**
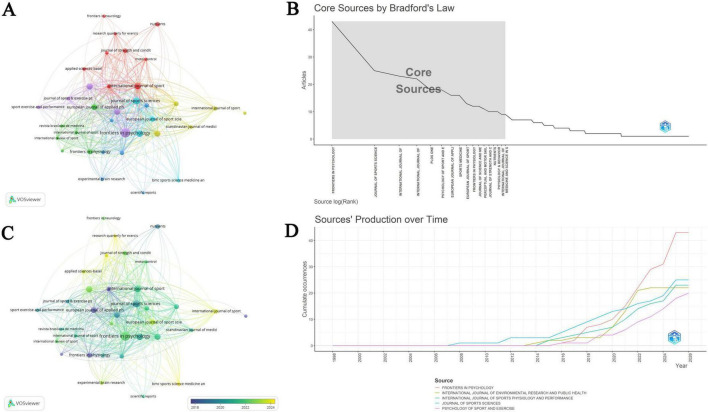
Analysis of journals. **(A)** Network visualization illustrating academic journals collaborations. **(B)** Core journals by Bradford’s law. **(C)** The overlay visualization of bibliographic coupling between academic journals. **(D)** Annual publication trends of the top five journals.

As presented in Table 2, *Frontiers in Psychology* and *International Journal of Environmental Research and Public Health* demonstrated the highest academic influence within this domain, both attaining an h-index of 14. *Frontiers in Psychology* exhibited the greatest publication output (43 articles), whilst *International Journal of Environmental Research and Public Health* recorded a substantial total citation count (1,055). These were followed by *Journal of Sports Sciences* and *European Journal of Applied Physiology*, each with an h-index of 13, publishing 25 and 16 articles, respectively, and accumulating 919 and 631 citations. This pattern persisted throughout the journal landscape, indicating a consistent positive correlation between journal h-index values and both publication productivity and citation impact. A total of 668 journals published articles on exercise and cognitive fatigue. Among these, 37 journals contributed more than 5 articles. Co-authorship analysis was visualized for these 37 journals, generating network visualizations of institutional collaboration and overlay mappings of inter-journal bibliographic coupling (Figures 5A,C). Bibliographic coupling analysis revealed that *European Journal of Applied Physiology*, *Frontiers in Physiology*, and *Medicine & Science in Sports & Exercise* were prolific in earlier periods. By contrast, *International Journal of Sport and Exercise Psychology*, *Sensors*, and *BMC Sports Science, Medicine and Rehabilitation* demonstrated heightened publication activity in recent years (Figure 5C). Figure 5B illustrates that the core zone comprised 17 high-quality journals, representing 4.98% of the total, which collectively published 279 articles in accordance with Bradford’s Law. Notable journals within this core zone included *Frontiers in Psychology*, *Journal of Sports Sciences*, *International Journal of Environmental Research and Public Health*, and *International Journal of Sports Physiology and Performance*, all characterized by elevated h-index values. The top five journals exhibited varying degrees of growth following 2015, with *Frontiers in Psychology* demonstrating the most pronounced increase in recent years, likely reflecting substantially enhanced research output in relevant domains (Figure 5D).

**TABLE 2:** Top 10 most influential journals.

Rank	Journal	h_index	TC	NP
1	Frontiers in Psychology	14	630	43
2	International Journal of Environmental Research And Public Health	14	1,055	22
3	Journal of Sports Sciences	13	919	25
4	European journal of Applied Physiology	13	631	16
5	International Journal of Sports Physiology and Performance	12	672	23
6	Sports Medicine	12	2,190	16
7	Frontiers in Physiology	10	593	12
8	Journal of Science and Medicine in Sport	10	372	12
9	PLOS ONE	9	503	18
10	Psychology of Sport and Exercise	8	263	20

TC, total citations; NP, number of publications.

### 3.5 Author keywords frequency and trend topics

At the author level, our analysis elucidated the relationship between publication metrics and academic influence (Table 3). The h-index and total citation count served as principal indicators of scholarly impact, whilst publication output reflected author productivity. ROELANDS B ranked foremost in h-index (16), with total citations of 1,506 and 1,428, and 328 relevant publications.

**TABLE 3:** Top 10 most influential authors.

Rank	Author	h_index	TC	NP
1	Roelands B	16	1,506	28
2	Meeusen R	14	1,371	16
3	Van Cutsem J	14	1,422	17
4	COUTTS AJ	12	1,302	13
5	Ferreira MEC	10	418	13
6	Bray SR	9	377	15
7	Marcora SM	9	2,148	10
8	De Pauw K	8	1,040	13
9	Díaz-García J	8	224	14
10	Fortes LS	8	286	10

TC, total citations; NP, number of publications.

**FIGURE 6:**
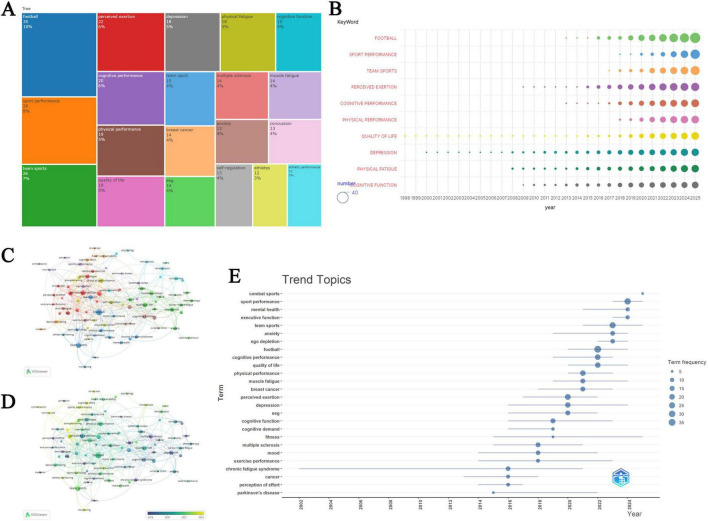
Analysis of keywords. **(A)** Tree map of the most frequent author keywords. **(B)** Annual publication trends of the top 10 keywords. **(C)** Network visualization illustrating of keywords. **(D)** The overlay visualization of keywords. **(E)** Trend topics in the research domain of Exercise and CF.

Analysis of the top 20 author keywords in the exercise and cognitive fatigue domain is presented in Figure 6A. The three most frequent author keywords were “football” (35 occurrences), “sport performance” (28 occurrences), and “team sports” (26 occurrences), constituting 10, 8, and 7% of all keywords, respectively. Figure 6B displays a bubble chart illustrating temporal trends in keyword frequency from 1998 to 2025. Overall, “quality of life” and “depression” represented persistent thematic concerns, whilst “football” and “sport performance” emerged as dominant focal points in recent years. A total of 1,890 distinct keywords appeared across articles on exercise and cognitive fatigue. Among these, 103 keywords occurred with a frequency exceeding 5. Keyword analysis was visualized for these 122 keywords, generating network visualizations and overlay mappings of keyword bibliographic coupling (Figures 6C,D).

Trend topic analysis revealed temporal evolution patterns within the research domain (Figure 6E). From 2002 to 2024, research themes exhibited substantial dynamism. “Chronic fatigue syndrome” emerged as the foundational theme in 2002, persisting for 19 years as the longest-spanning research orientation. Following a relative quiescence exceeding a decade, exercise performance assessment themes including “perception of effort” and “exercise performance” gradually coalesced into substantive research streams after 2014. During 2018–2021, the domain entered a rapid expansion phase, with thematic quantity tripling relative to the preceding period. “Perceived exertion,” “depression,” and “physical performance” constituted the core cluster of this era, whilst the emergence of “cognitive function” and “eeg” signified the establishment of neurophysiological mechanism research paradigms. During 2022–2023, a pronounced reorientation in research perspective occurred, with “football” (frequency = 35) and “team sports” (frequency = 26) becoming predominant hotspots. “Cognitive performance” (frequency = 20) appeared for the first time as an independent high-frequency theme, accompanied by mental health thematic clusters encompassing “anxiety” and “ego depletion,” reflecting a paradigmatic shift from individual clinical intervention toward comprehensive assessment of team athletic performance and psychological load. Frontier themes during 2024–2025 further converged upon “sport performance,” “mental health,” “executive function,” and “combat sports.” Notably, “mental health” became formally established as an independent research orientation, “executive function” represented the trend toward refined assessment of cognitive control mechanisms, and “combat sports,” emerging most recently in 2025, may anticipate emerging research interests in cognitive load research within antagonistic sports. It is noteworthy that “depression,” with 18 occurrences and an 8-year duration, functioned as a critical nexus bridging clinical rehabilitation and sport psychology, whilst the dual-mainline structure formed by “cognitive function” and “cognitive performance” underscored the central position of cognitive dimensions within sport science. The aforementioned evolutionary trajectory reveals a paradigmatic progression within this domain from pathological fatigue mechanisms toward competitive cognitive optimization, and from singular physiological indicators toward an integrated “brain-body-performance” model. This transformation reflects the profound influence of global sport scientization and comprehensive athlete health management demands upon academic inquiry.

### 3.6 Thematic evolution and strategic positioning of research frontiers

**FIGURE 7:**
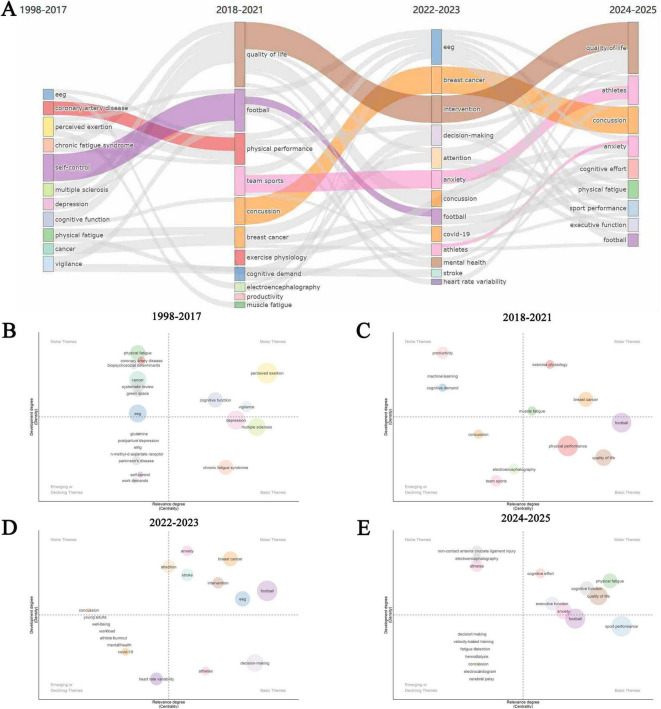
The evolution trend of keywords. **(A)** The changes and internal connections of keywords in different time periods. Thematic evolution within Exercise and CF in the periods 1998–2017 **(B)**, 2018–2021 **(C)**, 2022–2023 **(D)**, and 2024–2025 **(E)**.

Figure 7A illustrates the evolution and interrelationships of research themes across distinct temporal phases from 1998 to 2025. Nodes represent keywords, with size proportional to occurrence frequency, whilst edges denote co-occurrence relationships in the literature, with thickness reflecting co-occurrence intensity. To ensure balanced document distribution across phases, the study period was partitioned into four segments (1998–2017: *n* = 198; 2018–2021: *n* = 208; 2022–2023: *n* = 180; 2024–2025: *n* = 189), consistent with automated segmentation by Biblioshiny. Keyword networks within each segment demonstrate the shifting landscape of research priorities.

Thematic evolution analysis revealed a paradigmatic transformation from fragmented clinical descriptions (1998–2017) through consolidated team sports and brain injury research (2018–2021) toward neurophysiological monitoring and cognitive performance optimization (2022–2025). Early research exhibited parallel trajectories emanating from five thematic origins—“perceived exertion,” “depression,” “chronic fatigue syndrome,” “cognitive function,” and “physical fatigue”—without coherent clustering. During 2018–2021, a “football–quality of life–physical performance” triadic structure crystallized, generating four derivative branches: team sports, concussion, breast cancer, and EEG. The COVID-19 pandemic catalyzed heightened research activity in mental health, whilst “concussion” established its first self-stabilizing pathway, signifying the autonomization of sport-related brain injury research. During 2022–2023, thematic networks underwent progressive refinement, forming high-density chain pathways including “football → athletes,” “decision-making → cognitive effort,” and “EEG → executive function.” The incorporation of “heart rate variability” signaled deepening integration of physiological monitoring with athletic performance, with research perspectives shifting from sport modalities toward individual athletes. By 2024–2025, the research landscape converged, with “sport performance,” “cognitive effort,” and “executive function” emerging as terminal nodes. “Concussion” and “football” constituted high-stability bridging terms, whilst “quality of life” became established as the ultimate integrative endpoint. The overall evolutionary trajectory demonstrates a progression from dispersed clinical description through focused team sports and brain injury research toward a multidisciplinary integration era characterized by “physiological monitoring–cognitive assessment–performance optimization,” responding to imperatives for global sport scientization and comprehensive athlete health management.

Figures 7B–E illustrate the evolutionary trends of research themes within the exercise and cognitive fatigue domain across distinct temporal phases, with thematic maps generated through keyword clustering providing strategic overviews of domain dynamics. Each thematic map is partitioned into four quadrants: the horizontal axis represents centrality (degree of relevance to the domain), whilst the vertical axis represents density (degree of development within the domain). Centrality is computed as betweenness centrality within the co-occurrence network, quantifying the extent to which a node functions as a “bridge” between other nodes; higher values indicate more critical connective roles and stronger domain relevance. Density is measured through internal connection strength, specifically the ratio of aggregated co-occurrence edge weights to the maximum possible weight sum within the theme; elevated density values signify tighter internal associations and greater developmental maturity (Bornmann and Mutz, 2015; Dong et al., 2023) .

Strategic diagram analysis revealed structural transitions in research themes from 1998 to 2025. The early phase (1998–2017) was characterized by a “perceived exertion–cognitive function–vigilance” motor theme, with clinical disease themes occupying specialized quadrants and psychological regulation and neurological disease concepts situated in emerging zones, signaling the domain’s rapid progression toward integration of subjective perception, cognitive assessment, and clinical intervention (Figure 7B). The middle phase (2018–2021) featured “exercise physiology–muscle fatigue–breast cancer” as motor themes, with methodological innovation and cognitive demand analysis clustering in specialized themes, whilst brain injury monitoring and team sports concepts awaited integration, marking a transition from clinical rehabilitation toward performance optimization (Figure 7C). The recent phase (2022–2023) exhibited “anxiety–breast cancer–stroke–EEG–football–attention” as co-dominant motor themes, with the neurophysiological monitoring–mental state–sport context–cognitive function chain consolidated as the core engine; pandemic-related stress and athlete mental health remained in conceptual validation stages, with research accelerating toward neuro-psycho-motor integration (Figure 7D). The current phase (2024–2025) demonstrates “cognitive effort–physical fatigue–cognitive function–executive function–quality of life–anxiety” constituting motor themes, with the “brain–body–performance” integrative framework fused as the domain’s core driving force; sport injury prevention and neuro-monitoring technologies continue intensive development, whilst decision-making mechanisms, velocity-based training, and fatigue detection methods undergo paradigmatic renewal (Figure 7E). Collectively, these bibliographic patterns indicate the domain’s progression from “physiological mechanism description” toward a “cognition–execution–precision monitoring” integration stage, with motor themes providing neurophysiological–psychocognitive dual engines, specialized themes deepening injury and monitoring technologies, basic themes maintaining cross-domain support, and emerging themes pointing toward novel directions in training methodologies and clinical translation.

## 4 Discussion

### 4.1 Domain overview and bibliometric landscape of exercise and cognitive fatigue research

Bibliometric analysis of 820 publications (1998–2025) reveals a domain characterized by exponential growth (annual rate 1.84%), Anglo-European institutional leadership, and a journal landscape dominated by multidisciplinary psychology and sports science outlets (Figures 2–5). The following sections analyze the intellectual structure and thematic evolution underlying these descriptive patterns.

### 4.2 Temporal dynamics of research hotspots

Trending topic analysis illuminates the iterative progression of research hotspots within the exercise and cognitive fatigue domain. Influential themes first emerged in 2002, with subsequent evolution divisible into four distinct phases (1998–2017, 2018–2021, 2022–2023, 2024–2025) consistent with thematic evolution patterns. During 1998–2017, “chronic fatigue syndrome” functioned as the foundational theme with a 19-year duration, its 2002 debut preceding subsequent themes by 11 years and establishing the benchmark framework for pathological fatigue mechanism research. Co-occurrence of “perception of effort” with “Parkinson’s disease” and “cancer” indicated that subjective fatigue perception scale development and validation constituted core tasks, with samples predominantly restricted to chronic disease populations and research objectives focused on confirming concordance between subjective reports and objective physiological indices rather than exploring exercise-cognition causal mechanisms. The early appearance of “multiple sclerosis” signaled the incorporation of neurodegenerative disease cohorts into baseline observations, laying data foundations for subsequent longitudinal investigations. During 2018–2021, the co-occurrence network reveals a thematic convergence of “perceived exertion,” “EEG,” and “cognitive function” (Figure 6E). This clustering pattern coincides temporally with the global mental health crisis and may reflect a disciplinary response to growing demands for multidimensional assessment frameworks. Whether this bibliographic shift corresponds to genuine theoretical integration in primary research—or merely represents concurrent but independent research streams—cannot be determined from co-occurrence data alone. High-frequency occurrence of “perceived exertion,” “eeg,” and “cognitive function” demonstrated research scene expansion from clinical laboratories to athletic performance assessment, with attention directed toward neurophysiological monitoring and mental state interactions. Synchronous elevation of “depression” and “mood” indicated mental health outcomes incorporation into core metrics, whilst co-occurrence of “physical performance” and “exercise performance” reflected athletic performance testing becoming mainstream data collection methodology, providing technical support for large-scale exercise-cognition association studies. Thematic quantity surged from 4 to 12, with clinical themes (“breast cancer,” “muscle fatigue”) paralleling cognitive themes (“cognitive demand”), signifying research gravity shifting from singular fatigue description toward physiological-psychological-cognitive multidimensional mechanism exploration. During 2022–2023, team sports and cognitive performance optimization became dominant paradigms. Concentrated emergence of “football,” “team sports,” and “cognitive performance” indicated the field’s commencement of secondary integration of accumulated exercise-cognition data, validating cognitive performance models within team sport contexts. Synchronous incorporation of “quality of life” and “anxiety” demonstrated these outcomes becoming core endpoints, whilst first appearance of “ego depletion” reflected psychofatigue mechanism research deepening from physiological toward cognitive resource theoretical frameworks. Digital monitoring technologies and psychological-cognitive assessments registered explosive growth, signaling research gravity shifting from laboratory control toward authentic sport contexts and online intervention development. During 2024–2025, precision monitoring and executive function emerged as novel growth points. First appearance of “executive function” alongside “sport performance” and “mental health” indicated research commencement of utilizing specific cognitive components to parse causal directions between athletic performance and cognitive function, filling gaps in previous global cognitive assessment. Emergence of “combat sports” as a 2025 de novo theme signaled research scene refinement from team ball sports toward antagonistic combat disciplines, with attention directed toward neural performance under high-intensity intermittent cognitive-motor loading. Subjective scales and neurophysiological indices were retained as phenotypic anchoring tools, forming a “behavioral testing–EEG monitoring–executive function assessment” multidimensional evidence chain, portending the domain’s progression toward precision and specialization intervention new stages.

### 4.3 Convergence of keyword trajectories and thematic strategic positioning

Comprehensive comparison of keyword temporal evolution with strategic quadrant analysis of thematic trends reveals substantial concordance in phase demarcation, thematic attribution, and evolutionary logic. During 1998–2017, frequency peaks concentrated upon clinical fatigue-related terms including chronic fatigue syndrome, perception of effort, and cancer, corresponding to “motor themes” (perceived exertion, cognitive function, vigilance) and “basic themes” (depression, multiple sclerosis) in Figure 7B. This pattern elucidates early research characteristics dominated by pathological fatigue mechanisms and subjective perception measurement tool validation, with samples largely restricted to chronic disease populations and research objectives focused on establishing fatigue assessment benchmark frameworks rather than exploring exercise-cognition causal mechanisms. During 2018–2021, significant elevation occurred in perceived exertion, depression, cognitive function, and physical performance (thematic quantity surging from 4 to 12), consistent with “physiological-psychological-cognitive multidimensional integration” motor themes (exercise physiology, muscle fatigue) and “methodological innovation” specialized themes (machine learning, cognitive demand) in Figure 7C. This reflects rapid research transition from clinical laboratories toward athletic performance assessment, with neurophysiological monitoring (EEG) and mental health outcomes incorporated into core indicator systems. During 2022–2023, co-occurrence of football, team sports, cognitive performance, quality of life, and the anxiety-ego depletion chain precisely corresponded with “neuro-psycho-motor integration” motor themes (football, attention, EEG) and “pandemic stress” emerging themes (mental health, COVID-19) in Figure 7D. This indicates domain formation of a “team sports–cognitive performance–quality of life” closed-loop intervention research paradigm, with research gravity shifting from laboratory control toward authentic sport contexts. During 2024–2025, first aggregation of executive function, sport performance, and combat sports corresponded to “cognition–execution–precision monitoring” motor core (cognitive effort, executive function, physical fatigue) in Figure 7E, marking formal research entry into executive function refined assessment and specialized sport (combat discipline) precision intervention integration stages. Basic themes (football, physical performance, quality of life) and specialized themes (concussion, electroencephalography, athletes) demonstrated sustained stability in both keyword evolution and quadrant distribution, further validating intrinsic consistency within academic genealogies. Collectively, keyword trajectories and thematic strategic maps mutually corroborate, jointly delineating a four-stage evolutionary pathway: “pathological fatigue measurement → physiological-psychological integration → team sports optimization → executive function precision.” The concordance between keyword trajectories and thematic strategic maps indicates a four-stage bibliographic evolution: from pathological fatigue measurement, through physiological-psychological integration, to team sports optimization, and toward executive function precision. This observed pattern suggests shifting research priorities and emerging conceptual foci; however, it does not constitute evidence for actual theoretical consolidation or methodological improvement in primary studies.

### 4.4 Thematic evolution across strategic quadrants

Thematic evolution trend analysis enables identification of emerging research directions within the exercise and cognitive fatigue domain. Our focus centers upon thematic evolution during 2024–2025.

Themes including cognitive effort, physical fatigue, cognitive function, executive function, quality of life, and anxiety occupied the upper-right quadrant (Motor Themes), exhibiting elevated internal and external connectivity. This indicates these research directions have established relatively mature systems within their own domains whilst maintaining extensive cross-fertilization and synergistic interactions with other orientations, constituting current core research thematic directions with substantial outward diffusion capacity and future research potential.

The clustering of “cognitive effort” within motor themes during 2024–2025 (Figure 7E) indicates that this construct has achieved high centrality and density in the recent literature. This thematic prominence may reflect increasing primary research investigating how sustained attentional demands influence subsequent motor output, though bibliometric co-occurrence alone cannot establish causal directionality. Existing research demonstrates cognitive effort operates upon the motor system through two primary pathways: first, by elevating perceived exertion to reduce exercise motivation and persistence, with investigations demonstrating that high cognitive demand tasks significantly reduce participants’ benefit-cost evaluation ratios for exercise through heightened mental fatigue, thereby decreasing exercise selection likelihood (Harris and Bray, 2019); second, through direct impairment of specific athletic performance parameters, including repetition numbers in low-to-moderate intensity resistance exercise (de Lima-Junior et al., 2024), rate of force development, and running distance in intermittent endurance tests (Fortes et al., 2024; Silva et al., 2025). Notably, cognitive effort effects upon high-intensity anaerobic exercise and maximal strength performance remain controversial, with some investigations failing to detect significant impairment, suggesting exercise intensity may modulate cognitive fatigue’s negative effects (Campos et al., 2022) . Mechanistically, computational modeling has revealed cognitive effort accumulates fatigue sensations in real-time, with error-induced fatigue fluctuations exhibiting domain specificity, whilst individualized cognitive effort to exhaustion points does not necessarily impair subsequent vigorous exercise performance, challenging traditional “cognitive-physiological resource competition” hypotheses (Matthews et al., 2023; Holgado et al., 2025). Current research paradigms predominantly utilize standardized cognitive load induction through Stroop tasks and N-back tasks to elicit mental fatigue; however, ecological validity limitations and subjective-objective fatigue indicator dissociation remain methodological challenges (Claros-Salinas et al., 2013). Physical fatigue is primarily induced by high-intensity or prolonged physical activity, manifesting as decreased muscular function, altered physiological indices (e.g., heart rate variability, blood lactate levels), and elevated subjective fatigue; cognitive fatigue is induced by sustained cognitive tasks (e.g., Stroop tasks, N-back tasks), involving decrements in attention, executive function, and motivation (Mortimer et al., 2024; Yu et al., 2025). Multiple investigations have confirmed these fatigue types operate through distinct mechanisms: physical fatigue directly impairs task performance requiring strength, endurance, and movement precision (e.g., basketball shooting accuracy, jump height, change-of-direction speed), whereas cognitive fatigue predominantly affects performance through elevated ratings of perceived exertion and reduced psychological alertness, particularly in sports requiring decision-making and action anticipation (Yu et al., 2025). More intriguingly, when combined (dual fatigue), effects are not simply additive—some investigations demonstrate physical activity may alleviate cognitive fatigue’s negative effects, whilst cognitive load addition increases subjective mental fatigue without necessarily reducing actual physical work load (van As et al., 2021; Mortimer et al., 2024). Furthermore, the bibliographic separation of “physical fatigue” and “cognitive fatigue” into distinct thematic clusters (Figures 6C, 7E) parallels experimental literature suggesting differential neurophysiological correlates. Primary investigations have associated physical fatigue with autonomic nervous system modulation (Chen et al., 2023) and cognitive fatigue with dopaminergic pathway involvement (McMorris, 2020; Chen et al., 2023). However, the co-occurrence network reveals limited direct citation links between these thematic clusters, indicating that the literatures remain largely siloed despite their conceptual interrelation. Investigations demonstrate exercise effects upon cognitive function and executive function present bidirectional complexity. Acute aerobic exercise typically improves cognitive functions such as working memory, yet superimposed cognitive task demands may induce cognitive fatigue and negate exercise benefits (Kamijo and Abe, 2019). Conversely, high-intensity interval exercise or consecutive-day sprint training precipitates executive function decline, mental fatigue elevation, and mood fluctuation, whilst low-concentration oxygen-enriched air combined with exercise, brief mindfulness interventions, or simple leg movement (e.g., fidgeting) effectively alleviate cognitive fatigue and improve executive function (Costello et al., 2022; Fryer et al., 2022; Zhu et al., 2022; Zhang et al., 2025). Additionally, special populations such as breast cancer chemotherapy patients may improve self-reported cognitive function, cognitive fatigue, and executive function through exercise, yet hypoxic environment exercise may impair executive function through reduced dorsolateral prefrontal cortex activation (Ochi et al., 2018; Ren et al., 2022). Collectively, exercise effects upon cognitive function depend upon exercise intensity, duration, environmental conditions, and cognitive load accompaniment, necessitating careful weighing of acute benefits against potential fatigue accumulation costs. Existing research indicates exercise interventions positively influence quality of life in cognitive fatigue patients, though this effect is often indirectly mediated through cognitive fatigue alleviation. In cancer patient cohorts, supervised aerobic exercise significantly reduced cognitive fatigue in colorectal cancer chemotherapy patients, yet quality of life improvements were not observed (Zopf et al., 2022). In multiple sclerosis patients, physical activity combined with mindfulness or implementation intentions improved physical function and quality of life, with cognitive fatigue reduction correlating with psychological quality of life changes (Torkhani et al., 2021). Furthermore, a home-based online exercise program reduced cognitive fatigue by approximately 60% and significantly improved health-related quality of life physical function dimensions in postoperative breast cancer patients (Moulton et al., 2023). These findings suggest exercise effects upon quality of life may depend upon cognitive fatigue improvement degree, with variation across disease populations, necessitating further exploration of optimal exercise protocols for comprehensive quality of life enhancement. Anxiety maintains complex bidirectional relationships with exercise and cognitive fatigue. Trait anxiety predicts athlete psychological exhaustion, with high-anxiety athletes exhibiting stronger psychophysiological stress responses during competitive sports, including elevated mental fatigue levels and sympathetic overactivation, potentially precipitating sleep quality deterioration and recovery impairment (Tanguy et al., 2018). In concussion clinical characterization research, anxiety/mood is listed among five core clinical features, closely associated with cognitive/fatigue features, with female athletes scoring significantly higher than males on this dimension (Kontos et al., 2019; Stephenson et al., 2023). Additionally, anxiety symptoms co-occur with multiple fatigue states including cancer-related fatigue and chronic fatigue syndrome (Chan et al., 2013; Rha et al., 2019), and interact with sleep quality and depressive symptoms (Zhan et al., 2020; Huan et al., 2022). Anxiety-targeted interventions including mindfulness training, music intervention, and qigong practice have proven effective in alleviating mental fatigue and improving athlete cognitive performance and recovery states (Li et al., 2015; Wagner and Wieczorek, 2024; Pan et al., 2025b).

In the upper-left quadrant (Niche Themes), thematic terms including non-contact anterior cruciate ligament injury, electroencephalography, and athletes exhibited high external connectivity and low internal connectivity. This indicates that whilst these themes maintain relatively weak internal connections, they possess close associations with other research directions within the domain. Electroencephalography serves as a core neurophysiological tool extensively applied to monitor brain function alterations under athlete cognitive fatigue states, with investigations demonstrating that prefrontal cortex EEG theta power changes validate mental fatigue states and reveal associations with 20-kilometer cycling time trial performance decrements and elevated effort perception (Pires et al., 2018), and that wavelet analysis of mental fatigue effects upon low-intensity cycling tasks demonstrates enhanced desynchronization during pedal propulsion phases and enhanced synchronization during release phases in beta frequency bands (Proost et al., 2024). Simultaneously, non-contact anterior cruciate ligament injury constitutes a critical issue in sports injury prevention intersecting with cognitive fatigue research—investigations indicate mental fatigue significantly alters lower extremity biomechanical characteristics during stop-jump maneuvers, including reduced ankle dorsiflexion and knee flexion angles, increased ground reaction forces, and elevated knee abduction moments, thereby potentially enhancing non-contact anterior cruciate ligament injury risk (Zheng et al., 2025). Although these thematic terms exhibit relatively low internal connection density within specific research subgroups, they effectively bridge multiple research directions including athletic performance, neural mechanisms, and sports injury prevention as methodological tools (electroencephalography), research targets (athletes), or health outcomes (anterior cruciate ligament injury), reflecting the interdisciplinary integration characteristics of this domain.

In the lower-left quadrant (Emerging or Declining Themes), thematic terms including decision making, velocity-based training, fatigue detection, hemodialysis, concussion, electrocardiogram, and cerebral palsy exhibited low internal and external connectivity. This indicates that whilst these themes may have represented important research directions previously, their attention within relevant research domains declined during 2024–2025, gradually becoming marginalized research topics. Regarding decision making, investigations demonstrate mental fatigue significantly impairs technical performance and decision-making accuracy in football, basketball, and beach volleyball athletes, with such impairment closely associated with executive function decline and altered visual search behaviors (Sun et al., 2022; Pan et al., 2025a). Velocity-based training research reveals that high-intensity cognitive effort may reduce rate of force development without significantly affecting maximal strength gains or vertical jump performance, suggesting task-specific effects of mental fatigue upon resistance training (Fortes et al., 2024; Romagnoli et al., 2024) . For fatigue detection technologies, deep learning-based electrocardiogram and heart rate variability analysis methods have achieved 94.0% detection accuracy, providing technical support for real-time fatigue monitoring during exercise (Fan et al., 2025) . Hemodialysis research focuses upon exercise rehabilitation combined with calcitriol intervention for alleviating fatigue symptoms in end-stage renal disease patients, demonstrating multidisciplinary integrative therapeutic potential (Alishahi et al., 2024) . Concussion research has constructed clinical assessment frameworks encompassing cognitive-fatigue phenotypes, revealing associations between mental fatigue and landing biomechanics alterations alongside recovery trajectories (Shumski et al., 2023; Anderson et al., 2024; Preszler et al., 2024) . Cerebral palsy research endeavors to validate fatigue assessment tools across populations, confirming that modified mental fatigue scales exhibit favorable structural validity within this cohort (Bergqvist et al., 2020) . Collectively, these themes expand the boundaries of exercise and cognitive fatigue research, laying theoretical and empirical foundations for the development of precision intervention strategies.

In the lower-right quadrant (Basic Themes), thematic terms including football and sport performance exhibited high internal connectivity and low external connectivity, indicating these themes maintain substantial independence within the research domain and possess significant importance as autonomous research directions during 2024–2025. Cognitive fatigue research in football predominantly focuses upon negative effects of Stroop task-induced mental fatigue upon athletes’ technical performance, decision-making capacity, and running ability, with investigations demonstrating mental fatigue significantly reduces Yo-Yo intermittent recovery test distance, passing accuracy, and shooting speed in football players (Smith et al., 2016). Further investigations confirm mental fatigue primarily impairs technical rather than physical performance in small-sided football matches (Badin et al., 2016) . Additionally, research in rugby league demonstrates mental fatigue negatively correlates with post-match technical performance active engagement (Mariano et al., 2023). These investigations collectively reveal the significance of cognitive fatigue as an independent research theme within team ball sports, laying empirical foundations for continued domain development during 2024–2025.

### 4.5 Strengths and limitations

Strengths of this study include parallel retrieval from Web of Science and Scopus databases, inclusion of 820 publications, and integrated utilization of Bibliometrix and VOSviewer tools to achieve multidimensional bibliometric analysis. However, several limitations warrant acknowledgment: exclusion of specialized databases including PubMed and PsycINFO may result in incomplete coverage of peripheral disciplinary literature; citation lag and self-citation effects may influence emerging theme impact assessment; English-only inclusion introduces language bias; conceptual heterogeneity in search terms (e.g., interchangeable use of cognitive fatigue and mental fatigue) may precipitate relevant literature omission. Furthermore, the thematic evolution framework employed herein—based on keyword co-occurrence clustering and strategic diagram quadrant assignment—entails several inferential constraints that warrant explicit acknowledgment. First, temporal segmentation (1998–2017, 2018–2021, 2022–2023, 2024–2025) was determined by algorithmic document distribution balancing rather than theoretically meaningful historical breakpoints; alternative segmentation schemes might yield divergent thematic trajectories. Second, centrality and density metrics quantify network positional properties rather than conceptual importance or empirical validity; a theme may achieve high centrality through citation cartels or methodological bandwagon effects rather than genuine intellectual influence. Third, keyword co-occurrence does not imply causal or even logical connection between concepts; the co-appearance of “football” and “executive function” in the same article may reflect independent measurement variables rather than theoretically integrated constructs. Fourth, thematic maps are sensitive to synonym merging and threshold parameters; our synonym file construction and minimum occurrence thresholds (Section 2.2), while standard in bibliometric practice, inevitably impose interpretive frameworks that may obscure or artificially consolidate research traditions. Consequently, the thematic evolution narrative presented in Figures 7A–E should be interpreted as one plausible bibliographic organization among multiple possible configurations, indicative of shifting publication patterns rather than definitive disciplinary historiography. Future investigations may expand database scope, integrate machine learning topic modeling to enhance identification precision, and conduct longitudinal tracking validation of emerging themes.

## 5 Conclusion

Drawing upon 820 publications from Web of Science and Scopus databases, this study employed bibliometric methodology to systematically analyze the intellectual structure and developmental trajectory of the exercise and cognitive fatigue domain from 1998 to 2025. China and the United States dominated the research network with a combined 26.8% output share, whilst Vrije Universiteit Brussel, Universidade Federal da Paraíba, and journals including Frontiers in Psychology and Journal of Sports Sciences constituted core knowledge production bases. Electroencephalography technology, velocity-based training, and executive function assessment have supplanted traditional subjective scales to form an emerging methodological triad; football athletes, breast cancer chemotherapy patients, and COVID-19 convalescents serve as high ecological validity models for examining causal pathways between cognitive load and athletic performance; cognitive effort, physical fatigue, and quality of life function as key endpoints amenable to synchronous modification through exercise intervention, exhibiting highest betweenness centrality and internal density in strategic diagram analysis. Findings carry significant implications for exercise training practice: cognitive effort influences the motor system through dual pathways involving subjective perception and performance parameters; differentiated mechanisms of physical and cognitive fatigue provide theoretical foundations for precision fatigue management; whilst the establishment of executive function and quality of life as core endpoints signals paradigmatic shifting toward comprehensive athlete health management. However, whether this bibliographic convergence reflects actual methodological substitution in primary research—or merely parallel adoption without theoretical integration—requires further investigation. Future research should enhance ecological validity, explore individualized moderating effects, and construct multilevel theoretical models integrating physiological, psychological, and social factors, thereby responding to the profound demands of competitive sport scientization.

## Data Availability

The original contributions presented in this study are included in the article/supplementary material, further inquiries can be directed to the corresponding author.

## Footnotes

1
http://www.bibliometrix.org

2
https://www.VOSviewer.com
